# Applying an information literacy rubric to first-year health sciences student research posters*

**DOI:** 10.5195/jmla.2018.400

**Published:** 2018-01-02

**Authors:** Xan Goodman, John Watts, Rogelio Arenas, Rachelle Weigel, Tony Terrell

## Abstract

**Objective:**

This article describes the collection and analysis of annotated bibliographies created by first-year health sciences students to support their final poster projects. The authors examined the students’ abilities to select relevant and authoritative sources, summarize the content of those sources, and correctly cite those sources.

**Methods:**

We collected images of 1,253 posters, of which 120 were sampled for analysis, and scored the posters using a 4-point rubric to evaluate the students’ information literacy skills.

**Results:**

We found that 52% of students were proficient at selecting relevant sources that directly contributed to the themes, topics, or debates presented in their final poster projects, and 64% of students did well with selecting authoritative peer-reviewed scholarly sources related to their topics. However, 45% of students showed difficulty in correctly applying American Psychological Association (APA) citation style.

**Conclusion:**

Our findings demonstrate a need for instructors and librarians to provide strategies for reading and comprehending scholarly articles in addition to properly using APA citation style.

## INTRODUCTION

Information literacy instruction provides an essential foundation for students who are contemplating careers in the health sciences. Throughout their undergraduate experiences, health sciences students engage with health sciences and scientific literature in increasingly sophisticated ways. Evidence shows that first-year students entering university environments struggle with the content of scholarly and scientific literature [1–3]. These students also lack sophisticated research skills to help them navigate resources that are available through university libraries [1–5]. Thus, first-year health sciences students must build foundational skills to help them develop critical thinking and lifelong learning skills that will be valuable throughout their college careers and beyond [4, 5].

This study was designed to examine the abilities of first-year undergraduate health sciences students to select relevant and authoritative sources, follow and use American Psychological Association (APA) citation style for sources, and summarize selected sources to support their research topics for a final poster project. By characterizing the information literacy skills of first-year health sciences students, the authors aimed to guide librarians’ efforts to improve their information literacy instruction.

## METHODS

### Setting and population

This study focused on students enrolled in “Inquiry and Issues in Health Sciences” (HSC 100), a required first-year seminar for students who intend to major in the health sciences at the University of Nevada, Las Vegas. Students in this course often select a major in nursing, pre-dental, pre-medical, or other health sciences majors. HSC 100 is a high-enrollment gateway course that meets twice a week in a large auditorium-style classroom. The culminating assignment for this course is a research poster exploring a health sciences topic of the student’s choice. Each student individually develops a research topic, seeks scholarly sources addressing the topic, assembles the required elements of the project on a standard tri-fold poster board, and presents the research to the class during a large poster session on the final day of class.

To prepare students for finding the required research articles, librarians provide an hour-long instruction session with a lecture on locating scholarly research articles using library databases and Google Scholar. Librarians also discuss various characteristics of a scholarly article, including its purpose, general structure, and intended audience. Students are pointed to an online research guide, which includes links to recommended databases, citation information, and the lecture presentation materials from the instruction session.

### Data collection and preparation

This study was deemed exempt from review by the Institutional Review Board of the University of Nevada, Las Vegas (protocol #1311-4646M). The team—which included a health sciences librarian, teaching and learning librarian, and library technician—attended poster sessions each semester from fall 2013 to spring 2015. Each team member was assigned an area of the poster session from which to collect data. Using iPads provided by the library, team members took photos of each poster in their designated areas. We agreed that photos should be taken of each entry of the annotated bibliography as well one holistic photo of the poster to provide context for the annotations. After each poster session, we transferred photographs from the iPads to a password-protected file drive.

A graduate student assistant was hired to de-identify the images, removing all student information using Adobe Photoshop software. From the 6 semesters of poster presentations, we obtained images from 1,253 posters. A total of 413 posters had legible images and were analyzed using the sampling methods described below.

### Sampling of student work

Based on Oakleaf’s recommendations to avoid rater fatigue [6], we randomly selected approximately one-third of the legible subset of posters to form a representative sample. As we expected that each rater could carefully score 30 posters in 1 week, we scored one-third of the sample to prevent rater exhaustion and still provide a robust sample for scoring. Randomization was performed using the RANDOM.ORG integer generator by creating a series of integers from 1 to 413 with no repeats. Each integer corresponded to a folder number that represented a single poster. Ten randomly sampled posters were used for the rubric norming process, and 120 randomly sampled posters underwent formal scoring.

### Rubric design

In alignment with Mertler’s model for rubric development [7], we began developing the HSC 100 information literacy rubric by defining the learning outcomes of the assignment. The assignment required students to: (1) locate peer-reviewed research articles; (2) evaluate the sources for relevance to their topics; (3) summarize the sources, including their research methods and conclusion; and (4) cite the sources using APA style. To design the rubric, we reviewed guidelines for information literacy rubrics [6, 8] and settled on four performance levels (1–4) representing a range of student abilities to meet each of the four rubric criteria (Table 1).

**Table 1:** Information literacy rubric

Performance level	Competent	Developing	Beginning	Indeterminate or poor

Rating	4	3	2	1
Relevance	Clearly and specifically defines topic. Selected sources contribute directly to topic/argument/debate.	General topic is understood but may demonstrate a need to specify. Topic is covered generally, as opposed to in depth.	Sources peripherally touch on topic or go on tangents from main idea. Sources tease at topic without ever addressing it or answering a question.	Sources are of little to no significance or relevance to the topic.
Authority	Choice of material demonstrates a discerning eye for scholarly and non-scholarly sources.	Selection demonstrates a significant understanding of scholarly material but may include some questionable sources (e.g., op-ed, secondary source, magazine, WebMD).	Selections demonstrate significant obstacles in discerning appropriate sources. Sources may be too similar or ambiguous as to whether they come from scholarly sources.	Selections come from spurious sources (e.g., Wikipedia, blogs, forum posts). Sources use conjecture or anecdotal data.
Summary	Student demonstrates an ability to determine the strengths and weaknesses of sources or exactly what it answers or does not answer.	Student demonstrates competence in evaluating material but may overreach conclusions from the information.	Student demonstrates significant lack in comprehending the meaning of findings or how to apply findings correctly.	Uses information to reinforce unsubstantiated point. Pushes subjective or impartial narrative while citing information.
Citation	Correctly follows American Psychological Association (APA) format on all citations. Cites all sources. Knows information that is appropriate to cite (common knowledge vs. attribution).	Consistently uses APA format with little to no errors. Demonstrates competence but a need for improvement in discerning appropriate places to cite.	Citations show an inability to adhere to APA or a different citation format altogether. Student demonstrates significant lack of understanding as to where to place citations.	Has haphazardly selected or has no citations. Shows inconsistent or no use of APA.

### Inter-rater reliability

Before scoring posters using the rubric, we met to discuss the application of the rubric to student work and to determine inter-rater reliability. We practiced rating 3 posters together and achieved consensus on the guidelines for applying the rubric; these 3 posters were excluded from further analysis. To measure inter-rater reliability, we randomly selected 10 posters for each rater to score independently. We achieved a Krippendorff’s alpha [9] value of 0.853 among all 4 raters, which was sufficient to allow independent scoring [10].

### Analysis of posters

Each rater scored 30 posters within 1 week to prevent bias in scoring due to fatigue. Scores on each rubric criterion were summed to provide an overall score for each poster that ranged from 4 to 16. Scores were analyzed using SPSS software.

## RESULTS

We scored a total of 120 posters. Of the 4 rubric criteria, students scored lowest in the areas of summary and citation and highest in the areas of relevance and authority (Table 2).

**Table 2:** Poster scores

Performance level	Competent		Developing		Beginning		Indeterminate or poor			

Rating	4		3		2		1		Mean	

	n	(%)	n	(%)	n	(%)	n	(%)	n	(SD)
Relevance	62	(51.7%)	33	(27.5%)	22	(18.3%)	3	(2.5%)	3.2	(0.85)
Authority	79	(65.8%)	25	(20.8%)	13	(10.8%)	3	(2.5%)	3.5	(0.78)
Summary	41	(34.2%)	42	(35.0%)	33	(27.5%)	4	(3.3%)	3.0	(0.86)
Citation	38	(31.7%)	28	(23.3%)	48	(40.0%)	6	(5.0%)	2.8	(0.94)

## DISCUSSION

Our results suggested that first-year students, although new to using scholarly sources, had a good grasp on how to locate scholarly articles for an annotated bibliography assignment. Approximately half of the students were competent in their abilities to locate scholarly articles that were relevant to the topics they covered in their final posters. These findings demonstrate that these first-year students knew how to navigate library databases, Google Scholar, and possibly other Internet search engines to locate scholarly articles, which is consistent with previous studies [11–13]. Students also performed well in selecting scholarly peer-reviewed sources that were written by authors with expertise or authority related to their health topics, which is consistent with previous studies [12, 13].

Over half of the students demonstrated an early stage of developing proficiency with summarizing articles, suggesting that summarizing an article including all the required elements for an assignment is a complex skill, which is in agreement with previous studies [14, 15]. A final summary should be well written, be concise, and critically connect the scholarly articles to the student’s research topic. However, our students showed varying abilities in summarizing articles, perhaps because they were novices in both reading and summarizing scholarly articles. Therefore, librarians can provide instruction on how to identify parts of an article to help improve students’ summarization skills. Based on these findings, our library instruction team altered HSC 100 instruction to allow students more in-class time to practice reading scholarly articles, recognizing sections of an article, understanding the meaning of each section, and summarizing articles.

We found that first-year health sciences students struggled the most with citing articles, which is consistent with previous studies [16–19]. It is important for students to credit others’ ideas to avoid acts of intentional or unintentional plagiarism. Although students had access to an online guide with links to guidance on how to properly cite sources using APA style, we found that students needed more help in understanding how to cite scholarly articles. Citation is a complex skill that requires students to understand the parts of a citation to render correct output. Automatic citation generators might contribute to students’ inability to understand how to correctly apply a particular style to a citation [16, 18]. Librarians can help improve student performance by providing additional consultation appointments or tutorials to increase student proficiency with APA citation style.

In future research on undergraduate information literacy skills, researchers could conduct a longitudinal study to examine annotated bibliographies created by health sciences students at critical points throughout their undergraduate education, tracking individual students from a first-year seminar course through their capstone or culminating experiences. Such a research design could include refined rubrics designed to assess article summary, citation, and selection skills, with a targeted focus on students’ abilities to articulate the strengths and weaknesses of scholarly articles. It may also be valuable to perform a pre-assessment of student skills to draw stronger connections between the role of information literacy instruction and student outcomes.
